# Trends in Sri Lankan cause-specific adult mortality 1950–2006

**DOI:** 10.1186/1471-2458-14-644

**Published:** 2014-06-25

**Authors:** Chiranthika Vithana, Christine Linhart, Richard Taylor, Stephen Morrell, Syed Azim

**Affiliations:** 1 Ministry of Health, Colombo, Sri Lanka; 2 School of Public Health and Community Medicine (SPHCM), Faculty of Medicine, University of New South Wales (UNSW), Kensington (Main) Campus. Samuels Building, Level 2, Room 223, Botany St, Gate 11, Randwick Sydney, NSW, 2052, Australia

**Keywords:** Sri Lanka, Cause-specific mortality, Infectious diseases, Circulatory disease

## Abstract

**Background:**

Although all-cause mortality in Sri Lanka decreased significantly from 1950 to 1970, subsequent declines have been more modest with divergent trends by age and sex. This study investigates these trends through cause of death analysis for 1950–2006 in adults aged 15–64 years.

**Methods:**

Deaths were obtained from the World Health Organisation (WHO) mortality database for 1950 to 2003, and the Department of Census and Statistics Sri Lanka for 1992–95 and 2004–06 where WHO data was unavailable. Adult deaths were categorised by age (15–34 and 35–64 years) and sex into: infectious diseases; external-causes; circulatory diseases; cancers; digestive diseases; respiratory diseases; pregnancy-related; ill-defined; and other-causes. Cause-specific mortality rates were directly age-standardised to the 2001 Sri Lankan Census population.

**Results:**

Mortality declined in females aged 15–34 years by 85% over 1950–2006, predominantly due to sharp declines in infectious disease and pregnancy-related mortality over 1950–70. Among males aged 15–34 years the mortality decline was less at 47%, due to a rise in external-cause mortality during 1970–2000. In females aged 35–64 years mortality declined by 67% over 1950–2006, predominantly due to a sharp decline in infectious disease, ill-defined and other cause mortality over 1950–70. Among males aged 35–64 years, decline in mortality is evident to 1960 (19%) from decline in infectious disease mortality, followed by increased mortality from circulatory diseases and external cause mortality, despite continued decline in infectious disease mortality. All-cause mortality in males 35–64 years has stagnated since 1970, with fluctuating increases. Circulatory diseases were the leading cause of death among adults 35–64 years in 2002–06, with the male rate almost three times higher than females.

**Conclusions:**

Significant disparities are demonstrated in Sri Lankan cause-specific adult mortality by sex and age group for 1950–2006. Female mortality progressively declined while male mortality demonstrated periods of increase and stagnation. Among males aged 15–34 years this coincides with periods of civil conflict over 1970–2000. Among males aged 35–64 years the increased mortality from non-communicable disease and external causes are the main reasons for stagnation in all-cause mortality since the 1970’s.

## Background

Sri Lanka is an island nation of 65,610 square kilometres, with a population of approximately 19 million at the 2001 Census, consisting of mainly Sinhalese (74.5%); Sri Lankan Tamils (11.9%); Indian Tamils (4.6%); and Sri Lankan Moors (8.3%) [1]. Provisional data from the 2011 Census estimates the population to be around 20,278,000, with disaggregation by ethnicity yet to be released [2]. Following significant declines in mortality and increased life expectancy during the second half of the twentieth century, Sri Lanka came to be regarded as an impressive example of the population health improvements that could be achieved in developing countries with limited economic resources [3,4]. These achievements were attributed to social welfare policies and programmes implemented by the Sri Lankan Government, which included: free health care services from primary through to tertiary level; free education, also from primary through to tertiary level; and subsidised food distribution, namely rice [5-7]. By the early 1980’s Sri Lanka had significantly reduced mortality and achieved estimates of life expectancy approaching those of some high-income countries including Singapore, New Zealand and Ireland [4,8].

In recent decades, however, Sri Lanka has experienced an epidemiological transition that has seen morbidity and mortality from non-communicable diseases (NCDs), such as cardiovascular disease, increasing to rates higher than observed in many industrialised nations including the United Kingdom, the United States of America, Australia and France [9]. Despite commendable success in controlling pre-transitional conditions, including infectious disease, under-nutrition, and maternal and child morbidity and mortality, the major health challenge faced by Sri Lanka in the 21st century is developing effective community-based health promotion and disease prevention interventions to address the increasing prevalence and associated premature mortality from NCDs.

Previous studies of Sri Lankan mortality have primarily focused on trends in all-cause mortality, or cause-specific mortality for all age groups, and both sexes combined [10,11]. The present study analyses cause-specific mortality trends in Sri Lankans aged 15–64 years from 1950 to 2006 by age and sex. The present study investigates whether all-cause premature adult mortality in Sri Lanka has continuously declined over 1950–2006, as a consequence of control of pre-transitional conditions, or whether the decline has been interrupted at various ages in each sex by increases in other causes of death characteristic of the epidemiological transition.

## Methods

This study analyses sex-specific secular trends in cause-specific mortality over 1950–2006 in Sri Lankans aged 15–34 and 35–64 years.

### Data sources

Sri Lankan deaths by cause, sex and five-year age group were obtained from the World Health Organisation (WHO) mortality database for 1950–2003 [12], and from the Department of Census and Statistics (DCS) Sri Lanka for 1992–95 and 2004-06 [13] where WHO data was unavailable. Both sources derive their data from the Sri Lankan vital registration system where deaths are classified by a trained coder at the central Registrar General’s Department using scanned death declaration forms from the District Secretariat offices [14]. The Sri Lankan vital registration system classifies cause-specific mortality according to the International Classification of Diseases (ICD) schedule. Several revisions in ICD coding have occurred over 1950–2006 with Sri Lanka adopting ICD-6 in 1950, ICD-7 in 1969, ICD-8 in 1974, ICD-9 in 1980 and ICD-10 in 1997 [15]. Denominator populations by sex and five-year age group were obtained from the WHO database for 1950–1986 and from DCS Sri Lanka for 1987–2006 [12,13].

### Analysis

Cause-specific mortality data was categorised into; infectious diseases; external-causes; circulatory diseases; cancers; digestive diseases; respiratory diseases; pregnancy-related causes; and all remaining mortality categorised as other- and ill-defined causes. Any infectious diseases coded elsewhere in the ICD schedule were relocated to the infectious disease chapter to enable a more valid analysis of NCD trends, including mortality from acute and chronic rheumatic disease previously located in the circulatory disease chapter, and infectious diseases previously located in the respiratory and digestive disease chapters. The cancer and external-cause groups remained unaltered from the ICD classification.

**Figure 1:**
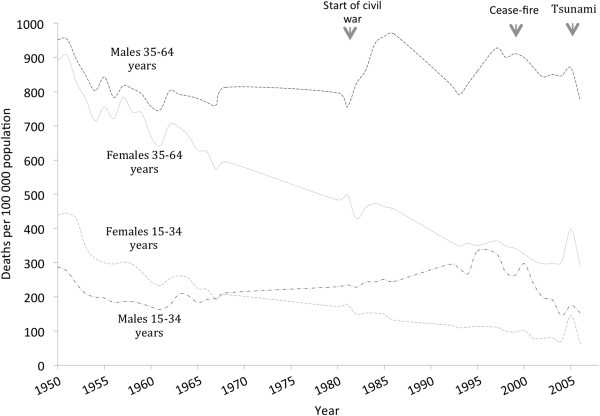
Sri Lanka, all-cause adult mortality, 15–34 and 35–64 years, 1950-2006*. *Directly age-standardised to 2001 Sri Lankan Census population. Origin of data: World Health Organisation statistical information system [12] 1950–1986 and 1997–2003; Department of Census and Statistics Sri Lanka [13] 1992–1995 and 2004–2006. No data available for 1969–79; 1987–91; 1996.

Mortality rates (per 100,000 population) were directly age-standardised to the 2001 Sri Lankan Census population to minimise confounding from changes in the underlying age-structure of the population. Results of analysis are presented by age, sex and period for all-cause mortality (Figure 1), and cause-specific mortality (Table 1). Periods in the Table 1 have been selected based on availability of data for each decade. Probable artefacts were identified in 1982 and 1996 when mortality in both age groups and sexes, and major causes, sharply changed. The most probable explanation is decreased registration, followed by its restoration, which in 1982 is likely to have been associated with the start of civil conflict. These periods are not included in the analysis*.* For periods where cause-specific mortality data were not available (1969–1979, 1987–1991 and 1996) rates were derived by linear interpolation between values of immediately adjacent years (Figure 1). Microsoft Excel 2010 was used for analysis.

**Table 1:** Sri Lanka, cause-specific adult mortality, 15–34 and 35–64 years, 1950-2006

	Cause-specific mortality rates^ and proportional mortality* ++									
	**Infect**	**Ext**	**Circ**	**Canc**	**Digst**	**Resp**	**Preg**	**Other**	**Ill-def**	**All**
**Males 15–34 years**										
1950-54	101 (47)	56 (26)	13 (6)	4 (2)	8 (4)	6 (3)	-	25 (12)	28 (12)	242
1960-64	43 (25)	64 (38)	22 (13)	6 (4)	7 (4)	3 (2)	-	25 (15)	15 (8)	184
1980-84	21 (10)	145 (66)	21 (10)	6 (3)	5 (2)	4 (2)	-	17 (8)	17 (7)	236
1992-96	16 (6)	222 (80)	17 (6)	5 (2)	7 (3)	3 (1)	-	7 (3)	19 (6)	296
2002-06	9 (6)	113 (70)	11 (7)	6 (4)	7 (4)	2 (1)	-	13 (8)	11 (6)	172
**Males 35–64 years**										
1950-54	294 (38)	84 (11)	141 (18)	40 (5)	43 (6)	28 (4)	-	139 (18)	122 (14)	890
1960-64	150 (22)	86 (12)	193 (28)	57 (8)	45 (6)	14 (2)	-	151 (22)	81 (10)	777
1980-84	91 (13)	145 (20)	242 (34)	64 (9)	34 (5)	36 (5)	-	102 (14)	120 (14)	836
1992-96	72 (11)	168 (25)	232 (34)	58 (8)	60 (9)	35 (5)	-	60 (9)	140 (17)	825
2002-06	58 (7)	162 (21)	231 (30)	75 (10)	93 (12)	46 (6)	-	114 (15)	59 (7)	837
**Females 15–34 years**										
1950-54	166 (47)	22 (6)	18 (5)	5 (1)	7 (2)	9 (3)	97 (27)	29 (8)	41 (10)	394
1960-64	65 (29)	29 (13)	25 (11)	7 (3)	5 (2)	4 (2)	47 (21)	43 (19)	24 (10)	249
1980-84	21 (14)	69 (48)	16 (11)	6 (4)	3 (2)	5 (3)	7 (5)	17 (12)	16 (10)	161
1992-96	13 (13)	54 (55)	12 (12)	5 (5)	2 (2)	3 (3)	2 (2)	7 (7)	14 (12)	113
2002-06	6 (7)	52 (63)	7 (8)	5 (6)	1 (1)	2 (2)	1 (1)	8 (10)	5 (6)	88
**Females 35–64 years**										
1950-54	285 (43)	23 (3)	85 (13)	54 (8)	24 (4)	27 (4)	39 (6)	146 (22)	154 (19)	836
1960-64	148 (26)	25 (4)	118 (21)	75 (13)	21 (4)	15 (3)	21 (4)	165 (29)	104 (15)	676
1980-84	55 (14)	37 (10)	107 (28)	76 (20)	11 (3)	27 (7)	3 (1)	76 (20)	85 (18)	478
1992-96	36 (13)	36 (13)	97 (35)	56 (20)	10 (4)	21 (8)	1 (0)	34 (12)	75 (21)	365
2002-06	22 (8)	49 (17)	84 (29)	68 (24)	9 (3)	21 (7)	0 (0)	51 (18)	29 (9)	336

^Cause-specific mortality rate per 100,000 population. *Cause-specific proportional mortality (%) excluding ill-defined; ill-defined as a proportion (%) of total deaths. Infect (infection); Ext (external); Circ (circulatory); Canc (cancer); Digst (digestive); Resp (respiratory); Preg (pregnancy-related); Ill-def (Ill-defined). ++Directly age-standardised to 2001 Sri Lankan Census population. Origin of data: World Health Organisation statistical information system [12] 1950–1986 and 1997–2003; Department of Census and Statistics Sri Lanka [13] 1992–1995 and 2004–2006. No data for 1969–79; 1987–91; 1996.

## Results

From 1950–2006 all-cause mortality for both sexes in Sri Lankans aged 15–34 years declined. Female mortality exceeded that in males from 1950 to 1966, after which male mortality exceeded female mortality, with the sex differential progressively widening until 2000. In adults aged 35–64 years, all-cause mortality declined to 1960 (19%), followed by stagnation since, although with fluctuating increases above the level in the 1960s. Female mortality (35–64 years) was lower than in males throughout 1950–2006, and the sex differential progressively widened (Figure 1).

*Infectious diseases* were the leading cause of mortality in the 1950’s for both age groups and sexes, with the highest mortality in males and females aged 35–64 years. A sharp decline in infectious disease mortality occurred during 1950–1970, followed by a continued decline for both age groups and sexes to 2002–06 (Table 1).

*External-cause mortality* increased among males during 1960–2000, with the sharpest increase in 15–34 year males in the 1990’s, followed by decline in both age groups after 2000. Among females, external-cause mortality remained less than half of the male rate through most of 1950–2006. Trends in external-cause mortality are associated with the start of the civil war in the early 1980’s and the cease-fire during 2001–05. The sharp mortality rise for both sexes and age groups in 2005 was due to the 2004 Boxing Day tsunami (26^th^ December 2004), with the associated mortality in females significantly higher than that in males. The late occurrence of the tsunami in 2004 resulted in the majority of the death registrations occurring in 2005.

*Circulatory disease mortality* in Sri Lankans aged 15–34 years remained low throughout 1950–2006 (both sexes). In adults 35–64 years it became the leading cause of death in both sexes from 2000, but almost three times higher in males than females (Table 1).

*Cancer mortality* in those aged 15–34 years remained low and stable throughout 1950–2006 (both sexes). In adults 35–64 years mortality increased over 1950–2006 in both sexes becoming the second leading cause of death in females aged 35–64 years in 2002–06 (Table 1).

*Digestive disease mortality* in those aged 15–34 years remained stable during 1950–2006 (both sexes). In adults aged 35–64 years digestive disease mortality declined then remained low in females from the 1980’s, while mortality among males increased from the 1990’s.

*Respiratory disease mortality* in those aged 15–34 years declined to 1960 then remained low in both sexes to 2002–06. Mortality fluctuated in females aged 35–64 years over 1950–2006, while mortality among males progressively increased from the 1980’s to 2002–06 (Table 1).

*Pregnancy-related mortality* halved between 1950–54 and 1960–64 in females aged 15–34 and 35–64 years, and then declined by a further 85% to 1980–84, with continued decline thereafter (Table 1).

*Other-cause mortality* (comprising all remaining deaths) varied from 3% to 29% of deaths during 1950–54 to 2002–06. The Other-cause proportional mortality was consistently higher (around 20%) in women 35–64 years than the other age/sex groups (Table 1).

*Ill-defined mortality declined* from 10-20% of total deaths 1950–54 to 6-12% in 2002–06.

## Discussion

Significant disparities exist in Sri Lankan cause-specific mortality trends by sex and age group over 1950–2006. Among adults aged 15–34 years, female mortality declined by 85% during this period, whereas the male mortality decline was considerably less at 47%. While sharp reductions in infectious disease mortality occurred in both sexes over 1950–70, there were significant rises in external-cause mortality in males over 1970–2000 (Figure 1 and Table 1). Among adults aged 35–64 years, female mortality declined by 67% over 1950–2006, whereas the male mortality decline was far lower at 19% with the main reasons being increased circulatory disease, other non-communicable disease and external cause mortality. Sri Lanka has experienced the epidemiological transition over recent decades with profound declines in pre-transitional conditions, including infectious disease and reproductive-related mortality in adults, and significant increases in transitional conditions, including circulatory disease and external causes (especially in males), and cancer (especially in females).

The most profound decrease in cause-specific mortality in Sri Lanka during 1950–2006 was infectious disease mortality, with death rates approximately halving between 1950–54 and 1960–64, and approximately halving again to 1980–84, followed by further subsequent declines to 2002–06. Sri Lanka’s achievements in lowering infectious disease mortality since the late 1940’s through prevention and treatment have been well documented in the published literature, and are largely attributed to the expansion and strengthening of communicable disease control interventions, especially against malaria, and a substantial increase in the availability of antibiotics [16,17]. Among males the sharp reduction in infectious disease mortality during 1950–70 was offset by a significant rise in external-cause mortality during 1970–2000, associated with protracted political violence which began in the 1970’s and continued to the cease-fire in 2001–05 [5,18]. External-cause mortality during this period was significantly higher among males than females. Estimates of mortality associated with civil unrest in Sri Lanka have been between 1,200 to 10,000 deaths from the 1971 insurrection waged by the Peoples Liberation Front; 12,000 deaths from the second insurrection in 1987–89; and over 60,000 deaths from the insurgency waged by the Liberation Tigers of Tamil Eelam from the 1970’s to 2000 [5]. However, no age- or sex-specific analysis is available [5].

Suicide trends in Sri Lanka are consistent with the rise in external-cause mortality from 1970–2000 among males in both age groups, and to a lesser degree among females 15–34 years. Gunnell et al. [19] reported that between 1950 and 1995 suicide rates in Sri Lanka increased 8-fold to reach one of the highest rates in the world at 47 deaths per 100,000 population, with the male rate almost three times higher than females. Abeyasinghe [20] calculated that suicide in Sri Lanka increased by 700% between 1960 and 1997, and Jayasekara [6] stated that more than half of all suicides in Sri Lanka occurred among people aged 15–29 years. By 2005 suicide rates had halved, with restrictions on the import and sale of toxic pesticides in 1995 (reportedly the most common suicide method) coinciding with suicide reductions in men and women of all ages [20].

External-cause mortality trends in both sexes and age groups declined from 2000 with the cease-fire, except for a sharp increase in 2005 due to the 2004 Boxing Day (26^th^ December 2004) tsunami which killed 31,000 Sri Lankans [21], with significantly higher mortality among females. Oxfam International suggested the reasons for greater female than male mortality included: women’s traditional role of caring for their husbands, children, and elderly relatives which kept them largely in and around their homes at the time of the tsunami; and women being physically weaker than men and less skilled in swimming or climbing trees [22].

Sri Lanka has experienced the epidemiological transition over recent decades with circulatory disease mortality the leading cause of death since 1960–64 in adult males 35–64 years. The rate in males remained twice as high than in females throughout 1950–2000, and almost three times as high since 2000. While the increasing burden of circulatory diseases, and NCDs in general, has been identified in Sri Lanka [9,23], few studies have noted significant gender disparities in morbidity and mortality by age group. Using data from the Registrar General’s Reports and DCS Sri Lanka, Nadarajah [10] calculated that among adults aged 15–44 years the male death rate (per 100,000 males) from diseases of the circulatory system increased by 78% between 1952–54 and 1970–72, while the female rate (per 100,000 females) decreased by 5% during the same period.

Strengths of the present study are that it presents cause-specific mortality rates where possible for 1950 to 2006, using the best data available. Mortality rates were age-standardised to overcome confounding due to changes in the age structure of the underlying population over time. In spite of efforts to obtain the most complete data available, there remain inherent weaknesses resulting from under-registration, particularly during periods of civil conflict. Mortality rates for periods where data was not available (1969–1979; 1987–1991; 1996) were derived by linear interpolation between values of immediately adjacent years (Figure 1). Probable artefacts were identified in 1982 and 1996 when mortality in both age groups and sexes, and major causes, sharply changed. The most probable explanation is decreased registration, followed by its restoration, which in 1982 is likely to have been associated with the start of civil conflict. These periods are not included in the analysis*.*

The main limitations of the present study are those that affect most cause of death analyses; that is, the validity and reliability of cause of death on death certificates and the quality of subsequent coding. Deaths in Sri Lanka are classified by a trained coder at the central Registrar General’s Department using death declaration forms from the District Secretariat offices [14]. A study carried out in 1996 assessing the quality and coverage of death certification in Sri Lanka found that 15.5% of the medical officers misclassified the underlying cause of death, with frequent use of ill-defined terms such as ‘cardiac arrest’ and abbreviations leading to misclassification in 26% of cases during this period [24]. However, misclassification of exact cause of death may not have a major impact at ICD Chapter level, as evidenced by validation studies [25].

## Conclusions

Sri Lanka has a history of implementing proactive policies to address the health needs of its population, and since the 1940’s has had a health system characterised by a strong primary care service with mechanisms to maximise equity and universal access at the local level. The structure of the Sri Lankan health system initially evolved to meet challenges including infectious diseases and maternal and child health care. Chronic illnesses have become the greatest cause of morbidity and mortality and the question has arisen how Sri Lanka can adapt its health system to deal with these new epidemics through reorientation of provincial health services to prevent NCD and meet the needs of the increasing number of people with these conditions.

## Competing interests

The authors declare they have no competing interests.

## Authors’ contributions

CV conceptualised the study, collected data and performed analyses. CL performed analyses and drafted the manuscript. RT edited the manuscript and performed analyses. SM edited the manuscript and performed analyses. SA performed analyses. All authors read and approved the final manuscript.

## Pre-publication history

The pre-publication history for this paper can be accessed here:

http://www.biomedcentral.com/1471-2458/14/644/prepub

## References

[B1] Department of Census and Statistics Sri Lanka Brief analysis of population and housing characteristics [http://www.statistics.gov.lk/PopHouSat/PDF/p7%20population%20and%20Housing%20Text-11-12-06.pdf]

[B2] Department of Census and Statistics Sri Lanka Census of Population and Housing 2011. Population of Sri Lanka by district. Preliminary Report (Provisional) – 1 2012 Colombo: Department of Census and Statistics

[B3] Caldwell JC Routes to low mortality in poor countries Popul Dev Rev 1986 12 2 171 220 10.2307/1973108 21174865

[B4] Balabanova D McKee M Mills A ‘Good Health at a Low Cost’ 25 Years On. What Makes a Successful Health System? 2011 London: London School of Hygiene & Tropical Medicine

[B5] Samaranayake G Demographic trends and political violence in Sri Lanka Sri Lanka J Popul Stud 2002 5 31 45

[B6] Jayasekara RS Community nurses: an urgent need Nurs Health Sci 2001 3 101 104 10.1046/j.1442-2018.2001.00073.x 11882185

[B7] Rajapaksa LC Oration of the College of Community Physicians 2010. Addressing health inequality – then and now Journal of the College of Community Physicians of Sri Lanka 2011 16 1 1 7

[B8] World Bank Life expectancy at birth country profiles 2006 [http://data.worldbank.org/indicator/SP.DYN.LE00.IN]

[B9] Abeywardena MY Dietary fats, carbohydrates and vascular disease: Sri Lankan perspectives Atherosclerosis 2003 171 2 157 61 10.1016/S0021-9150(03)00157-6 14644383

[B10] Nadarajah T The transition from higher female to higher male mortality in Sri Lanka (Popul Dev Rev 1983 9 2 317 325 10.2307/1973055

[B11] Langford CM Sex differentials in mortality in Sri Lanka: changes since the 1920s J Biosoc Sci 1984 16 399 410 6470022 10.1017/s0021932000015212

[B12] World Health Organisation Statistical Information System Detailed data files of WHO mortality, population and live births database 2008 [http://www.who.int/whosis/en/]

[B13] Department of Census and Statistics Sri Lanka Population and housing vital statistics 2008 [http://www.statistics.gov.lk/page.asp?page=Population%20and%20Housing]

[B14] World Health Organisation Status of mortality statistics of the country: Sri Lanka 2011 [http://s3.amazonaws.com/zanran_storage/www.searo.who.int/ContentPages/6493086.pdf]

[B15] Department of Census and Statistics Statistical abstracts 2010 2010 [http://www.statistics.gov.lk/abstract2010/Pages/index.htm]

[B16] Meegama SA The Mortality Transition in Sri Lanka Determinants of Mortality Change and Differentials in Developing Countries – the Five-Country Case Study Project 1986 New York: United Nations 5 32

[B17] Dissanayake L Epidemiological transition in Sri Lanka Sri Lanka J Popul Stud 2000 3 59 66

[B18] Ministry of Defence Sri Lanka Humanitarian Operation Factual Analysis July 2006 – May 2009 2011 July Colombo: Ministry of Defence

[B19] Gunnell D Fernando R Hewagama M Priyangika WDD Konradsen F Eddleston M The impact of pesticide regulations on suicide in Sri Lanka Int J Epidemiol 2007 36 1235 1242 10.1093/ije/dym164 17726039 PMC3154644

[B20] Abeyasinghe R Suicide Rate Community Survey of Suicide and Attempted Suicide: Presidential Report N.d

[B21] Disaster information management system Sri Lanka Tsunami deaths [http://www.desinventar.lk/DesInventar/index1.jsp]

[B22] Oxfam International The tsunami’s impact on women, Oxfam Briefing Note March 2005. Oxfam International [http://www.oxfam.org/sites/www.oxfam.org/files/women.pdf]

[B23] Engelgau M Okamoto K Navaratne KV Gopalan S Prevention and Control of Selected Chronic NCDs in Sri Lanka. Policy Options and Action 2009 World Bank: Washington, DC

[B24] Fonseka WAAP A Study in the Quality and Coverage of Death Registration in a District of Sri Lanka 1996 Postgraduate Institute of Medicine: Colombo

[B25] Carter KL Hufanga S Rao C Akauola S Lopez AD Rampitage R Taylor R Causes of death in Tonga: quality of certification and implications for statistics Population Health Metrics 2012 10 4 1 15 doi:10.1186/1478-7954-10-4. [http://www.pophealthmetrics.com/content/pdf/1478-7954-10-4.pdf] 22390221 10.1186/1478-7954-10-4 PMC3378436

